# Out of hours care: a profile analysis of patients attending the emergency department and the general practitioner on call

**DOI:** 10.1186/1471-2296-11-88

**Published:** 2010-11-15

**Authors:** Hilde Philips, Roy Remmen, Peter De Paepe, Walter Buylaert, Paul Van Royen

**Affiliations:** 1 Department of Primary and Interdisciplinary Care of the University of Antwerp, Belgium (PICA-UA), General Practice, Universiteitsplein 1, Geb R, 3de verd., B-2610 Wilrijk, Belgium; 2 Department of Emergency Medicine, Ghent University Hospital, De Pintelaan 185, B-9000 Ghent, Belgium

## Abstract

**Background:**

Overuse of emergency departments (ED) is of concern in Western society and it is often referred to as 'inappropriate' use. This phenomenon may compromise efficient use of health care personnel, infrastructure and financial resources of the ED. To redirect patients, an extensive knowledge of the experiences and attitudes of patients and their choice behaviour is necessary. The aim of this study is to quantify the patients and socio-economical determinants for choosing the general practitioner (GP) on call or the ED.

**Methods:**

Data collection was conducted simultaneously in 4 large cities in Belgium. All patients who visited EDs or used the services of the GP on call during two weekends in January 2005 were enrolled in the study in a prospective manner. We used semi-structured questionnaires to interview patients from both services.

**Results:**

1611 patient contacts were suitable for further analysis. 640 patients visited the GP and 971 went to the ED. Determinants that associated with the choice of the ED are: being male, having visited the ED during the past 12 months at least once, speaking another language than Dutch or French, being of African (sub-Saharan as well as North African) nationality and no medical insurance. We also found that young men are more likely to seek help at the ED for minor trauma, compared to women.

**Conclusions:**

Patients tend to seek help at the service they are acquainted with. Two populations that distinctively seek help at the ED for minor medical problems are people of foreign origin and men suffering minor trauma. Aiming at a redirection of patients, special attention should go to these patients. Informing them about the health services' specific tasks and the needlessness of technical examinations for minor trauma, might be a useful intervention.

## Background

Overuse of emergency departments (ED) is of concern in Western society and it is often referred to as 'inappropriate' use [1-6]. Patients assess their medical problems with worries and interpretations in their own context and may decide to seek help independently from referral or triage systems [7,8]. Although there is some consensus of doctors and nurses concerning the perception of 'emergency', important differences were found between the perception of patients and clinical staff [9,10]. Patients' perceptions of an emergency do not necessarily correspond with clinical interpretations made by health care providers [11]. What is or is not an 'emergency' can lead to different interpretations of 'appropriate' and 'inappropriate use' of ED.

Inappropriate use may compromise efficient use of health care personnel, infrastructure and financial resources of the ED [12]. Inefficient use also threatens timely treatment of serious medical conditions at the ED [13,14]. The opinion to redirect patients, however, is hampered by the discrepancies in appreciation between consumers, health care providers and financial backers as to the value of primary and secondary care services. Therefore the top down approach alone is insufficient as a solution. An extensive knowledge of the experiences and attitudes of patients and their choice behaviour is necessary. Services must pay attention to this knowledge to align out of hours care to people's preferences, in order to attract patients to the most efficient service [15].

We therefore, in a prospective study, compared populations of patients during out-of-hours at both secondary care services (emergency departments, EDs) and primary care services (general practitioners (GPs) on call). The aim was to quantify the patients and socio-economic determinants associated with choosing the GP on call or the ED. We also detailed reasons that patients mentioned for choosing a particular service.

## Methods

### Context

Belgian health care is characterized by free entrance to primary, secondary and tertiary care facilities. There is no gatekeeper role of general practitioners (GP) and no need for referral [16]. Physicians are most often paid on a 'fee for service' basis. Patients have obligatory medical insurance by which certain medical care is reimbursed. Out of pocket payment accounts for approximately 25% of health expenses [16]. For primary care, patients pay directly, while for secondary care, patients receive billings afterwards. At the time of the study co-payment systems at the ED were not compulsory and not in common use. Patients can be registered with a GP of their choice, but this is not obligatory to have access to all health care facilities. In Belgium, almost 99% of the population is covered with compulsory health insurance [16,17].

Providing 24 hours coverage is a legal obligation of GPs in Belgium [18]. GPs organise out-of-hours care in rotation systems. This service is organised by local general practitioner organisations. In these small scale organisations, GPs on call usually work from their private practices. Most of the local GP organisations use a phone number which immediately leads to the out-of-hours care facility. Patients have to find out for themselves which GP is available and where the practice is located. Prior telephone contact is not necessary; patients can walk in without appointment. There is no telephone triage; no consultation over the telephone is performed. Patients can come to the doctor's practice or ask the GP for a home visit [16]. Since 2003, in some regions in Belgium, the first general practitioner cooperatives (GPC) emerged.

### Materials

Data collection was conducted simultaneously in 4 large cities in Belgium (Antwerp, Ghent, Brussels, Charleroi). All patients who visited EDs or used the services of the GP on call during two weekends in January 2005 (Saturday 12 AM until Sunday 12 AM) were enrolled in the study in a prospective manner.

Directors of hospitals and primary care services were individually informed of the project and their participation was secured. The GPs on call and the services in the hospitals were regularly contacted by the principal investigator. Ethical approval was acquired for all services.

A semi-structured questionnaire was developed, based on literature, and piloted for this study. It comprised 6 domains and 39 questions. Senior medical students were trained to interview the patients at the various data collecting sites. They performed face-to-face interviews at the ED and telephone interviews with the GP patients after the doctor's visit. At the ED patients were asked to participate at the moment of entrance and data were collected immediately thereafter. As GP services were in many cities, and offered by more than one GP per region, we decided to collect data from these services by phoning immediately on the data of visit. GPs asked all patients whether they were willing to participate. If they agreed, the telephone number of the patient was registered in order to be contacted by the interviewer after the GP consultation.

For each patient the following data were collected about the consultation: demographic information (sex, age, postal code), date and hour of consultation, the Reason For Encounter (RFE), the diagnosis and whether or not subsequent hospitalisation was necessary. Also the manner by which they came to the medical service (self referral, physician's referral, ambulance, other) was registered. To assess the process of choice we also asked how they found the telephone number and address of the service, who made the decision to seek help at that service, what was their knowledge concerning the payment system, whether there had been earlier contacts with out- of-hours services and whether they had considered looking for help elsewhere. At the end of the interview the socio-economic status (family, nationality, language, income/financial situation, insurance) was registered. Patients who refused to participate were only asked for their characteristics (age, sex) and the RFE. When possible we also assessed the doctor's diagnosis and whether the patient was hospitalised or not after the doctors' examination. Data of non-participants were only used to assess case-load but not for further analysis.

After data collection, the researchers used ICPC2 to recode RFE and diagnosis. The variable 'minor trauma' was collected by searching the data manually and adding the code A80 when trauma was mentioned in the RFE. When a A80 code in the RFE was combined with a S18 (skin lesion) or an ICPC2 code concerning contusions and abrasions in the diagnosis, we included the case as 'minor trauma'.

### Data collection and analysis

Data were analysed using SPSS 16.0. We compared absolute numbers of contacts for each ICPC2 chapter between ED and GP contacts. Due to missing data concerning diagnosis in the data of Brussels and Charleroi, we restricted the descriptive analysis for the variables RFE and diagnosis to the data of Antwerp and Ghent.

We used uni-variant analysis with odds ratios (OR) and 95% confidence intervals (CI) where applicable. Nominal variables were compared with chi^2^-tests, whereas Mann Whitney tests were applied for the comparison of mean ages.

Binary logistic regression analysis with service choice (GP or ED) as the dependent variable, was used to compare patient and socio-economic determinants between both patient populations, computing odds ratios with their 95% CI. The choice of the determinants, relevant for this multivariate analysis was based on literature [4,19,20].

### Ethical approval

Ethical approval of this study was given by the Ethical Committees of the Universities of Antwerp, Ghent and Leuven: A04-77.

## Results

### Descriptive

A total of 1970 patients contacted one of the services and were eligible for inclusion at the four sites. 359 (18.2%) patients refused to participate. Reasons for refusal were documented in 27 (0.07%) cases: patient died (n = 2), the patient is an unaccompanied child (n = 19) or the patient was not able to participate (n = 6). 1611 patient contacts were suitable for further analysis, 640 in the GP population and 971 ED users. Main patient characteristics are listed in table 1.

**Table 1:** main patient characteristics at the GP services and the ED

	GP	ED
Gender (% men)	289/638 (45.3%)	492/968 (50.8%)

Mean age	35.7 years, SD 45.9	32.2 years, SD 23.3

Registered with a GP (% yes)	584/638 (91.5%)	754/967 (78.0%)

Used ED at least once during past 12 months (% yes)	185/634 29.1%)	379/960 (39.5%)

Employed (% yes)	354/622 (56.9%)	487/955 (51.0%)

First language Dutch or French (both national Belgian languages) (% yes)	580/639 (90.8%)	747/968 (77.2%)

Nationality:		
- Belgian	526/640 (82.2%)	642/968 (66.3%)
- African	20/640 (3.1%)	129/968 (13.3%)
- Other	94/640 (14.7%)	197/968 (20.4%)

Refusal rate of study participation, was significantly lower in the GP visitors (GP: 113 refusals (15%), ED: 246 refusals (20%)). The mean age (33.6 y, Standard Deviation (SD) 34.2) of the participants (N = 1611) was not significantly different from the mean age of the non-participants (N = 359) (38.0 y, SD 24.2) (P > 0.05). Men were more likely to refuse participation than women did (refusals: male N = 177 (58.8%), female N = 124 (41.2%))(p < 0.01). The relative numbers of subsequently hospitalised patients were significantly higher in the nonparticipants group compared to those in the participants group (hospitalised non-participants N = 64/225 (28.4%), hospitalised participants N = 206/1461 (14.1%)) (p < 0.01). The mean age of the patients that visited the GP on call is 35.7 (SD 45.9) years, which is significantly higher than the population at the ED (32.2 y, SD 23.3) (p < 0.05).

In the next part of this results chapter, we will focus only on the group of patients who participated (n = 1611).

The item 'diagnosis' was missing in 49.4% of cases in the GP group (N = 640). In the ED group only 3.8% of this data were missing (N = 971). Therefore we limited the descriptive part on this specific item to the databases of Ghent and Antwerp, where registration of 'diagnosis' was performed as planned in the study design. Table 2 shows RFE and diagnosis chapters in both services. For the diagnosis, chapters L 'musculoskeletal' (21.6%) and S 'skin' (17.3%) were the most prominent at the ED services, while R 'respiratory' (36.8%) and D 'digestive' (20.2%) were most prominent at the GP services.

**Table 2:** Absolute numbers of patients visiting the ED or the GP with Reason For Encounter (RFE) and Diagnosis according to ICPC2 chapters (database of Ghent and Antwerp)

ICPC2 chapter	RFE			Diagnosis		
	GP service	ED	Total	GP service	ED	Total
Missing	2	1	3	14	2	16
General and unspecified	96	83	179	31	52	83
Blood, blood forming organs	1	0	1	1	4	5
Digestive	68	62	130	68	60	128
Eye	3	13	16	1	11	12
Ear	9	5	14	13	4	17
Circulatory	6	8	14	7	25	32
Musculoskeletal	33	119	152	27	106	133
Neurological	19	26	45	10	17	27
Psychological	6	17	23	7	19	26
Respiratory	72	47	119	124	56	180
Skin	17	58	75	21	78	99
Endocrine, metabolic, nutrition	0	0	0	0	3	3
Urological	2	6	8	8	8	16
Pregnancy, child-bearing, family planning	0	0	0	1	1	2
Female genital	0	3	3	1	2	3
Male genital	2	2	4	2	2	4
Social problems	1	0	1	1	0	1

**Total**	**337**	**450**	**787**	**337**	**450**	**787**

**Table 3:** Chi2 analysis of trauma and non-trauma related RFE between men and women at the ED

ED (N = 971)	Trauma	Non-trauma	
Male	165	327	492

Female	131	345	476

Total	296	672	968

Chi^2 ^= 4.124, p = 0.0423

OR = 1.329, 95% CI: 1.010 - 1.749

**Table 4:** Chi2 analysis of trauma and non-trauma related RFE between men and women at the GP services

GP (N = 640)	Trauma	Non-trauma	
Male	32	257	289

Female	46	303	349

Total	78	560	638

Chi^2 ^= 0.655, p = 0.4185

OR = 0.820, 95% CI: 0.507 - 1.327

In the group of patients who decided to consult the GP (N = 640), 54 (8.4%) patients were not registered with a GP. In most cases the patient or a family member recommended calling the GP (93.2%). In this group of patients (N = 640), 105 (16.4%) initially considered going to the ED but decided to call the GP. 185 (28.9%) of the GP patients reported using the ED at least once during the past 12 months.

In the ED group (N = 971), 213 (21.9%) patients were not registered with a GP. In 86.6% of the cases (n = 841), the decision to go to the ED was taken by the patient or by a family member. In 8.0% of the cases (n = 78) someone else gave the advice to visit the ED (friends, neighbours, ...). Of this group (this question was answered by N = 681), 86 (12.6%) patients contacted the GP on call before going to the ED. The question of by whom they were referred to the ED was answered by 968 participants. In 618 cases (63.8%) patients reported going to the ED on their own initiative. Other referral possibilities were: referred by their own family physician (n = 67, 6.9%), by the GP on call (n = 57, 5.9%) or by a specialist doctor (n = 48, 5.0%). 126 were brought in by ambulance (n = 99, 10.2%) or police (n = 27, 2.8%).

On Chi^2 ^analysis, we found that men are more likely to seek help at the ED for minor trauma, compared to women. (OR = 1.329, 95% CI: 1.010-1.749) This difference is not significant at the GP services (OR = 0.820, 95% CI: 0.507-1.327).

People at the ED were asked why they decided to seek help at the ED. In order of absolute numbers the reasons are shown in table 5.

**Table 5:** Reasons for seeking help at the ED

Question: 'Why did you decide to seek help at the ED?' (more answers possible) (N = 971)	
**Reason**	**Absolute number of patients who checked the box (%)**

Accessibility	140 (14.4%)

Competence of personnel	110 (11.3%)

Proximity	107 (11.0%)

Open 24/7	88 (9.1%)

No knowledge of GP on call	70 (7.2%)

Family doctor not available	50 (5.1%)

No need for an appointment	39 (4.0%)

Not wanting to disturb the GP on call	26 (2.6%)

No need for immediate payment	10 (1.0%)

Of the 971 patients who visited the ED in our study, 379 (39.3%) had used the ED during the past 12 months at least once, 48 (4.9%) of them more than 3 times.

### GP or ED? A binary logistic regression analysis

We used binary logistic regression analysis with the use of the service (ED or GP) as dependent variable (GP being the reference category). Our best fitting model is described in table 6. We used 11 independent variables in the equation and six of them contributed significantly. Determinants that steered the choice in favour of the GP on call are: being female, having a family doctor and speaking Dutch or French (both national languages in Belgium). Determinants that advanced the choice for the ED are: being male, having visited the ED during the past 12 months at least once, speaking another language than Dutch or French, being of African (sub-Saharan as well as North African) nationality and lack of any medical insurance. Age, educational level and employment were not significant in this regression model.

**Table 6:** OR with 95% CI of independent variables in the equation with the choice for ED or GP as dependent variable (GP is the reference category, an OR > 1 is in favour of the ED)

	P value	OR	95,0% C.I. for OR	
			
			Lower	Upper
**Sex male (female)**	**0,049**	**1,249**	**1,001**	**1,559**

**Not registered with GP (Yes)**	**0,000**	**2,696**	**1,856**	**3,916**

**Did not visit the ED past 12 months (Yes)**	**0,001**	**0,675**	**0,533**	**0,855**

Education: No diploma or primary school	0,064			
Secondary school	0,870	0,972	0,691	1,367
University or High school	0,098	0,726	0,496	1,061

Age category (> 60 y)	0,339			
0-14 y	0,918	1,021	0,693	1,503
15-59 y	0,283	1,211	0,854	1,716

**Language (other than Dutch/French)**	**0,006**			
**French**	**0,001**	**0,491**	**0,317**	**0,761**
**Dutch**	**0,007**	**0,522**	**0,326**	**0,836**

Unemployed (Employed)	0,844	0,973	0,744	1,274

**Nationality (Belgian)**	**0,000**			
**African Sub-Saharan**	**0,008**	**3,726**	**1,400**	**9,914**
**North African**	**0,001**	**2,885**	**1,513**	**5,501**
Turkish	0,164	1,891	0,771	4,638
Other nationalities	0,436	0,859	0,585	1,261

**No medical insurance (Yes)**	**0,032**	**3,231**	**1,106**	**9,442**

Constant	0,000	10,859		

(Significant determinants are in bold)

'Income' (missing in 49.7% of cases) and 'family situation' were entered into the model but did not change the results significantly. Adding interaction terms 'nationality*language' or 'age*sex' did not ameliorate the model significantly either.

## Discussion and Conclusions

In this prospective study we compared profiles of 1611 patients at EDs and GP out-of-hours services in urban areas. Determinants for choosing a service were gender, having a family GP, having used the ED at least once during the past 12 months, language, nationality and having medical insurance.

According to table 2, musculoskeletal problems were the most frequent RFE and diagnoses at the ED. When keeping in mind that most RFE and diagnoses in ICPC-chapter S 'skin' are wounds or other traumatic skin lesions, we count 14.8% in the RFE at the GP and 39.3% at the ED that can be categorised as '(minor) trauma'. The same results are found for diagnoses: respectively 14.2% and 40.9%.

### Limitations of the study

Some limitations of this study need to be addressed. We had to deal with the absence of strict catchment areas of both ED and GPs on call. Due to health service organisation in Belgium, people can seek help wherever they choose. As the areas are not well defined, numbers of GP contacts and contacts at the ED do not necessarily cover all patients seeking urgent care and are not necessarily adding up to one hundred percent of medical consumption. For this reason we have to be careful in our conclusions concerning socio-economic minority groups at the ED, which may have come from the broader catchment areas, and this may lead to over interpretation of this particular group of patients.

We lacked information on diagnosis in approximately half of the GP cases, due to under-registration of these data in Charleroi and Brussels. Nevertheless, we compared our results to other studies and found very similar results in studies in France, Sweden and The Netherlands, therefore we presume satisfying validity of our data [21-24].

We managed to obtain information on the income of patients in 50.3% of all cases. Including this variable in the binary logistic analysis leads to a less valid model and was therefore omitted. Because we assume that income and other socio-economic factors influence the patient's choice, it was rather unfortunate to have missing data on this item. In former research socio-economic factors have variable influence on choice behaviour, therefore it would have been very interesting to make conclusions about those items in this setting [25-27]. Future research using 'Geographic Information Systems (GIS)' describing socio-economic factors regionally, might elicit its role on choice behaviour [28-30].

As severity of the medical problem was not included in the questionnaire, we have to take into account that we may not compare the reasons for seeking help at either one service in a valid way, for severity is a confounding factor. We may not conclude on 'appropriate' or 'inappropriate' use based on these findings, neither was this the scope of this study. We missed data on income. For this reason we intend to perform a new study in a qualitative design, in which it is more feasible to assess income and other socio-economic determinants.

### Findings

Men are more likely to seek help at the ED, often with ICPC codes relating to minor trauma (OR for male patients seeking help for 'minor trauma' versus female patients: OR = 1.329, 95% CI: 1.010 - 1.749). This confirms results of former research in which specifically young men rather seek help at the ED for minor trauma, suggesting that they appear to link their problem to technical examinations [31]. The most frequently mentioned reasons for choosing the ED are similar to findings in a questionnaire study in the Netherlands [32]. As the results of our study are similar, this indicates that this group is relatively free to choose, whether the GP appears to take the role of a gatekeeper or not.

People who used the ED during the past 12 months tend to return to the ED, whereas people who being registered with a GP, tend to seek help in primary care during out of hours. This confirms that people tend to choose the service which they are already acquainted with, as we have shown in a questionnaire study in the general public [2,31,33-35]. On the other hand, as we did not ask about the seriousness of the medical problem, another possible explanation could be that patients, who have visited the ED during the past 12 months, have more serious illnesses than other patients or suffer complications of former and/or chronic illnesses. Until now literature describes a 'returning behaviour' to the service patients know, further research has to take the seriousness of the complaint and patients history into account, to clarify its role in the choice behaviour of the patient.

Patients of foreign nationality presented themselves significantly more at the ED, hence bypassing the GP services. Cultural identity has been suggested as one indicator for different behaviour in the health system [26]. As those patients are acquainted to the healthcare system of their country of origin, they have less knowledge about the accessibility and organisation of out-of-hours services in other countries. Therefore, one can imagine that the GP services are, due to their structure, not accessible enough, as information of the services is not communicated in their language. Different types of organisation exist; in some regions GPs organize out of hours services at GP cooperatives, whereas other regions switch every weekend between GPs on call in a certain sequence. Perhaps the GPs, who work from their private practice, are sometimes difficult to locate or harder to reach.

Although financial aspects are not significant in our model, for this part of the community they might be more critical. The fee for service at the GP service and direct payment, might act as a patient selector [36]. This finding needs further investigation to explore reasons for this phenomenon. A qualitative approach can be used to explore how this specific population can be reached and how health care can be organised to minimize disparities.

In our setting 39.7% of all enrolled patients used the GP out of hours care and 60.3% the ED. Of all ED users 63.8% went to the ED without any referral. These figures might be subject to the health system. In other West European countries e.g. the Netherlands, where GPs are gate keepers and patients cannot easily attend a medical service without referral or telephone contact this percentage of direct ED referral is 43% [32,37]. Compared to research similar to ours, in The Netherlands and the UK, the number of ED visitors is much higher in Belgium than it is elsewhere [32]. Another explanation for this phenomenon could be the lack of any kind of telephone triage as it exists in other countries. In Belgium patients not only have free choice of medical services, but also free access. There is no need for any telephone contact before entering care facilities. This excludes steering choice behaviour by telephone triage in the current health care system in Belgium [38,39]. Implementation of triage systems in the future and research as to whether this might be a solution to redirect patients is therefore necessary.

One critical determinant is whether the patient has medical insurance [27]. Also in our study, people who do not have any medical insurance tend to go to the ED rather than to the GP. This finding could be explained by the current situation in this country where patients at the ED do not pay immediately and receive an invoice later on, while patients who go and see the GP need to pay directly. Studying socio-economic influences requires specific research, focusing on those regions where different minority groups are found. More research needs to be done concerning the influence of socio-economic factors as a driver for patient choice.

## Conclusion

In this, and in another study made by our group, we found that, in general, patients prefer the type of out-of-hours service that they know and have experienced [31]. A large proportion of patients at the ED do report having a GP, thus encouraging people to have a GP would probably not directly influence behaviour during out of hours. Two populations that distinctively seek help at the ED for minor medical problems are people of foreign origin and young men suffering minor trauma. Therefore, taking care of minorities in society by informing them about the possibilities of medical services could help to reallocate patients to the appropriate service. Also informing young people about the needlessness of technical examinations for most injuries and the availability of GPs during out-of-hours, could redirect patient streams, without diminishing quality of care. More research needs to be done concerning the influence of socio-economic factors as a driver for patient choice.

## Competing interests

The authors declare that they have no competing interests.

## Authors' contributions

PH contributed to the study design, data gathering, analysis and writing the text. RR contributed to the study design, data gathering and reviewing the text. DPP contributed to the study design, data gathering and reviewing the text. BW contributed to the study design, data gathering and reviewing the text. VRP contributed to the study design and in reviewing the text.

All authors read and approved the final manuscript.

## Pre-publication history

The pre-publication history for this paper can be accessed here:

http://www.biomedcentral.com/1471-2296/11/88/prepub
